# Supplementary material for: Low temperature near-field fingerprint spectroscopy of 2D electron systems in oxide heterostructures and beyond
Source: Nat Commun. 2025 May 13;16:4417. doi: 10.1038/s41467-025-59633-1 (PMC12075614; doi:10.1038/s41467-025-59633-1)
Supplement: Supplementary file 1 — Supplementary Information [file 41467_2025_59633_MOESM1_ESM.pdf]

Low temperature near-field fingerprint spectroscopy of 2D electron systems in oxide heterostructures and beyond

Julian Barnett[1], Konstantin G. Wirth[1], Richard Hentrich[2], Yasin C. Durmaz[2,3], Marc-André Rose[4], Felix Gunkel[4], and Thomas Taubner[1]*

Affiliations:

[1] I. Institute of Physics (IA), RWTH Aachen University, 52074 Aachen, Germany
[2] attocube systems AG, 85540 Haar, Germany,
[3] Department of Physics, Ludwig Maximilians University of Munich, 80799 Munich, Germany
[4] Peter Grünberg Institute (PGI-7) and Jülich-Aachen Research Alliance (JARA-FIT), Forschungszentrum Jülich, 52428 Jülich, Germany

*taubner@physik.rwth-aachen.de

Supplementary Information

S1: Comparison to previously published results

The main text discusses that previous publications of s-SNOM on the LAO/STO 2DEG were limited by the availability of light sources. Two cases were differentiated in Figure 1d: a) to investigate at higher frequencies (> 930 cm^{-1}),[1] where high-intensity light sources such as CO_2-laser or QCLs were available but the sensitivity of s_2/s_2^{Au} to changes of the dielectric function is much lower, or b) to investigate the phonon near-field resonance of STO,[2] where the scattering efficiency is higher but the influence of the 2DEG is mostly visible as additional damping via its contributions to the imaginary part of the dielectric function. Near-field fingerprint spectroscopy via self-referencing, as presented in this paper, allows for a much higher sensitivity of the s-SNOM signal to the electronic properties of the 2DEG. However, the scattering efficiency (absolute scattering amplitude) at the positions of the peaks is very low. As a result, the peaks will be lost if signal-to-noise of the light source is too low. To visualize this, published results from Synchrotron measurements of the same samples are compared directly with the new measurements shown in this publication.

Figure S1a shows the absolute scattering amplitude s_2 of the conducting LAO/STO sample, measured with a commercial s-SNOM (Neaspec GmbH) in nanoFTIR setup with synchrotron illumination from the Metrology Light Source (MLS) at Physikalisch-Technische Bundesanstalt (PTB) Berlin.[3] The storage ring was operated in a mode characterized by a low horizontal emittance, therefore leading to both a low beam size and a low beam divergence, making this mode particularly suited for s-SNOM measurements, due to high illumination power at the tip apex.[4] The black and red curve show two consecutive measurements at the same position, indicating the reproducibility of the measurement. In the spectral range between 700 and 750 cm^{-1}, the right flank of the near-field resonance peak is visible, whereas the signal at higher frequencies (above 750 cm^{-1}) is very low, as expected from theory (cf. Figure S2). Zooming in by a factor of 10 on the vertical axis (Figure S1b) shows that the overall reproducibility above 750 cm^{-1} is poor due to low signal-to-noise, which does not allow for self-referencing in this spectral region, as dividing by small numbers with high variation leads to a near-field fingerprint spectrum that consists of erroneous peaks.

Figures S1c and S1d present comparable measurement data used in this publication (cf. Figure 2 of main text), related to the region of highest sensitivity $\delta s_n/\delta\varepsilon$ (red-shaded area, cf. Figure S2). The reproducibility of individual measurements is much better, which results in reproducible normalized

spectra, as shown in the main text. Thus, the availability of new light sources (cf. Table 1) allows for the previously impossible utilization of near-field fingerprint spectroscopy to investigate small perturbations to the dielectric function in the vicinity of zero-crossings of Re[ε].

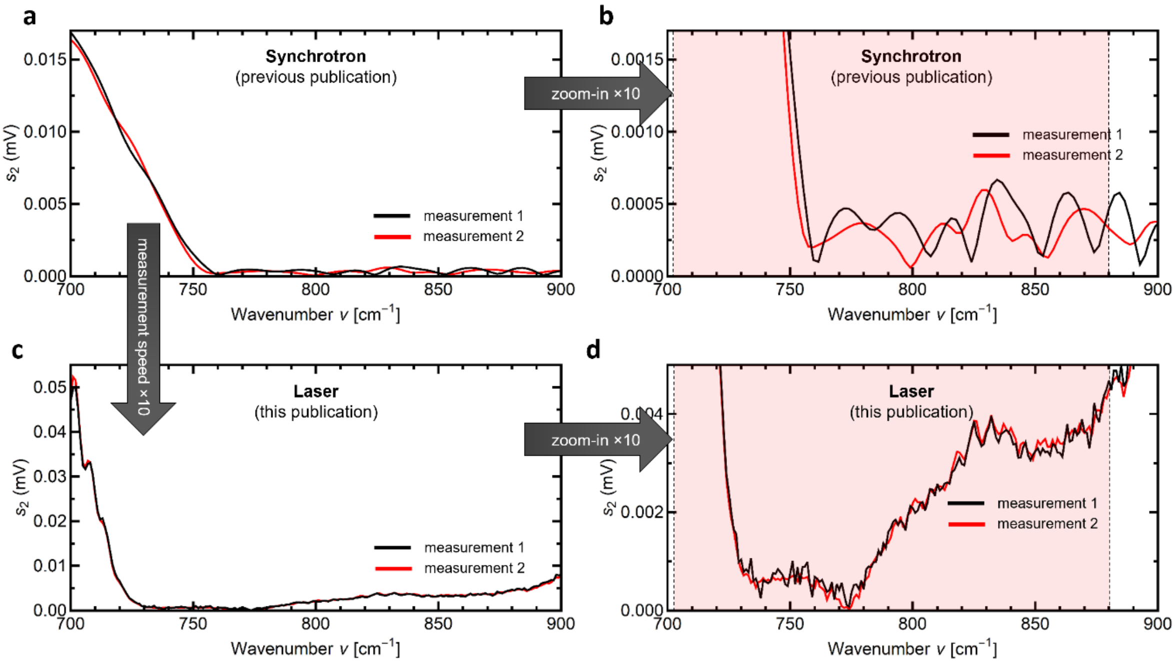

Figure S1: Comparison of measurements to previously published results. **a** Fourier-transformed scattering amplitude s_2 of two consecutive nanoFTIR measurements with Synchrotron illumination (see text for details), used in the previous publication.[2] **b** Zoom-in by a factor of ×10 on the vertical axis. **c** Scattering amplitude s_2 of two consecutive s-SNOM measurements with Laser illumination (see main text for details). The measurement speed was increased by approximately a factor of ×10 compared to measurements in Subfigure S1a. **d** Zoom-in by a factor of ×10 on the vertical axis. The red-shaded area denotes the previously identified region of highest sensitivity $\delta s_n/\delta\varepsilon$ (cf. Figure S2).

Table S1: Comparison of typical light sources used in SNOM.[5–7]

light source	spectral width [cm^{-1}]	tuning range [cm^{-1}]	est. spectral irradiance at focus [W/cm^2/cm^{-1}]
thermal source	4000	-	9×10^{-4}
very broadband laser	400	700-2500	$0.1\text{-}2 \times 10^{-2}$
synchrotron	2000	-	
broadband laser	75	500-2200	2-37
tunable laser (this paper)	3	625-2000	1,100
QCL	1	850-2800	17,000

S2: Generalized near-field fingerprint spectroscopy

The findings on near-field fingerprint spectroscopy presented in the main text are an advanced application of s-SNOM, that is new in the reported frequency range. The working principle of s-SNOM is based on coupled electromagnetic near-fields between tip and sample at optical frequencies. Assuming constant tip properties and tip-sample distance, the scattering signal is mostly dependent on the dielectric properties of the sample, introducing a frequency dependence via the dielectric function $\varepsilon(\nu)$. Figure S2a shows a generalized coupling function of the Au-normalized scattering amplitude s_2/s_2^{Au}, which depends on the real (x-axis) and imaginary part (grey scale) of ε. While s_2/s_2^{Au} converges for highly positive and negative values of Re$[\varepsilon]$, different behavior can be observed between values of +20 and -20. For low imaginary part (light grey curve), a high scattering amplitude is visible at slightly negative Re$[\varepsilon]$ (“near-field resonance”), while a minimum in scattering amplitude occurs at Re$[\varepsilon] \approx 1$. Thus, changes to ε of the sample result in non-linear changes of the scattering amplitude, with the highest sensitivity (maximum slope) usually found around Re$[\varepsilon] \approx -1$. This can be seen in the zoom-in in Figure S2b, where the range of maximum slope is highlighted in red. Decreasing Re$[\varepsilon]$ by a small amount (blue-green arrow), e. g. by introducing free charge carriers to the material, leads to a change in s_2/s_2^{Au} that is strongest in this range.

Figure S2c shows the dielectric function of a bulk STO single crystal (blue) in the range of the highest-frequency phonon mode. Additionally, a small shift of -0.3 to Re$[\varepsilon]$ is presented in green, much better visible in the zoom-in in Figure S2d, which shows the range around the LO frequency (zero-crossing) of the STO phonon. The red-shaded area again indicates the region of highest s-SNOM sensitivity, linked to Figure S2b via dashed lines. The resulting s-SNOM scattering amplitude is shown in Figure S2e, which can be directly compared to Figure 2f of the main text. In this case, s_2/s_2^{Au} is shown for bulk STO (blue curve) compared to the slightly decreased Re$[\varepsilon]$ (dashed green curve), resulting in changes I) and III) due to the perturbation, similar to the description of the conducting and insulating sample in the main text. However, change II) of Figure 1f is absent (greyed out), as it is a specific feature of the layered LAO/2DEG/STO stack. This shows that adding layers can lead to additional peaks in the fingerprint spectrum, which helps to disentangle different contributions to the s-SNOM signal.

Referencing the perturbed (changed ε) to the unperturbed case (unchanged ε) results in the normalized spectrum (solid green curve) shown in Figure S2f. Here, two peaks arise from the changes I) and III), that are characteristic for the perturbation to the dielectric function of the material. As these peaks are located in the frequency range where s_2/s_2^{Au} is most sensitive to changes of ε (red-shaded region), this near-field “fingerprint” is ideally suited to track slight variations of the optical properties of the material.

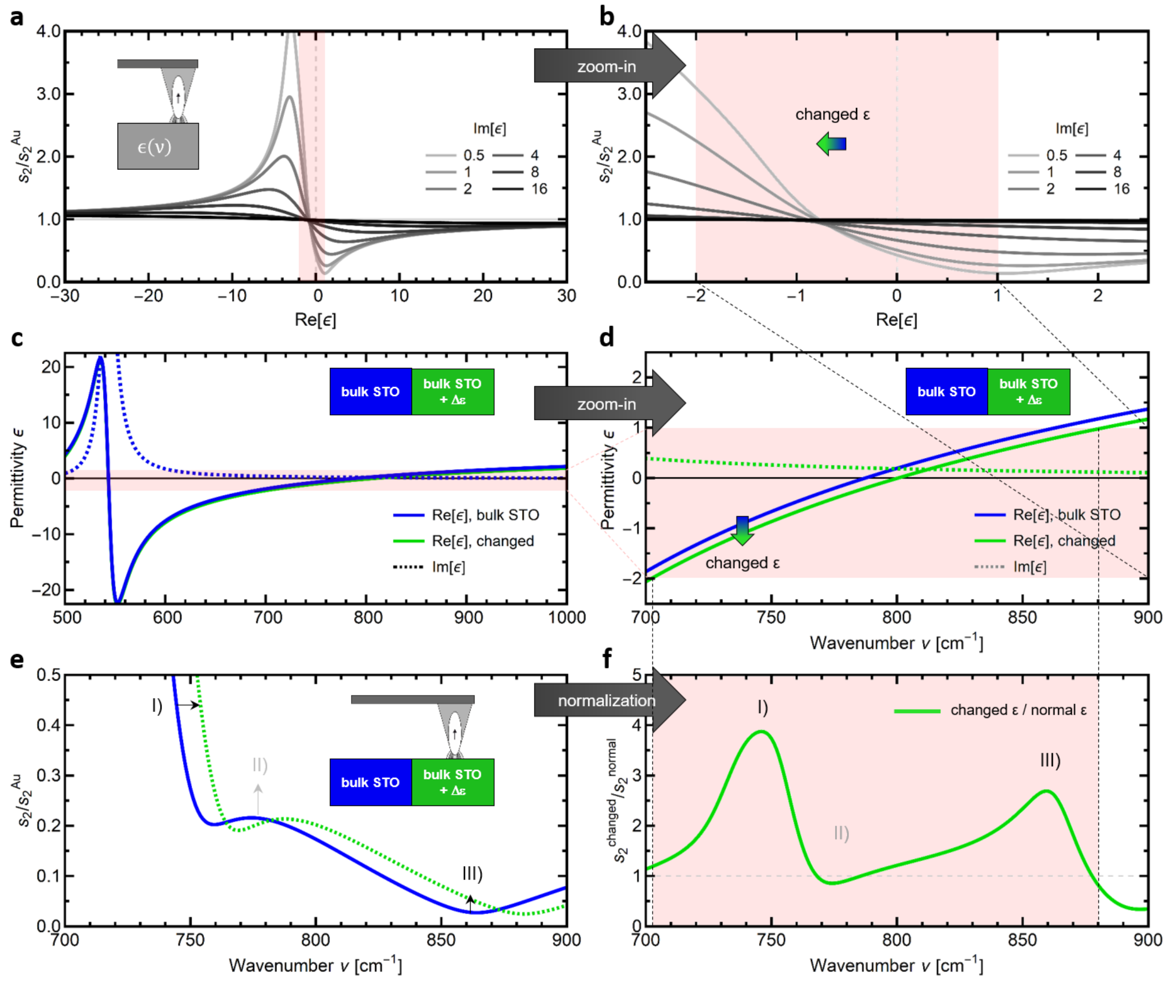

Figure S2: Working principle of fingerprint spectroscopy (simulations) for arbitrary perturbations $\Delta\varepsilon$ of a bulk material. **a** Coupling function of s-SNOM, i. e. the dependence of the scattering amplitude s_2/s_2^{Au} on the sample dielectric function for the surface of a bulk material. For low imaginary part (light grey), a pronounced near-field resonance (peak) is visible at slightly negative values of Re[ε], which yields the highest absolute scattering signals. **b** Zoom-in of the coupling function, showing the influence of the slightly changed dielectric function on s_2/s_2^{Au} by shifting 0.3 to the left (blue-green arrow). The red shaded region indicates the range of Re[ε] where the change of the scattering signal is strongest (maximum slope). **c** Real (solid line) and imaginary part (dashed line) of the dielectric function of bulk STO (blue), showing the highest frequency phonon mode; a perturbation $\Delta\varepsilon$ = -0.3 (green) introduced to the real part is barely visible on this scale. **d** Zoom-in of the dielectric function close to the LO-frequency (zero-crossing), showcasing the shifted real part (blue-green arrow). **e** Resulting near-field amplitude spectrum s_2/s_2^{Au} of bulk STO (blue) compared to the result of the slightly changed Re[ε] (dashed green). Changes I) and III) are highlighted (cf. Figure 1f of the main text), while change II) is not present (greyed out). **f** The normalization procedure results in two characteristic peaks (cf. Figure 1g of the main text), with position, height and shape depending on the magnitude of the change as well as on the original scattering spectrum of the bulk material. The missing peak II) (grey label) is a feature of the layered system and thus not present in the bulk case shown here.

At this point, it should be emphasized that the behavior described here is universally applicable to any bulk or layered material that exhibits a zero-crossing of Re[ε] at low Im[ε], such as the LO frequency of a phonon mode. As was shown in the main text, adding layers can lead to the appearance of additional peaks, depending on the relative position of the zero-crossings of Re[ε] of each material. Furthermore, the perturbation $\Delta\varepsilon$ as shown here typically results from adding free charge carriers, making this a model case for doped oxides, topological insulators and conducting 2D materials.

S3: Simulation parameters

Figure 1b and following: The bulk dielectric functions of $LaAlO_3$ (LAO) and $SrTiO_3$ (STO) were calculated using the Berreman-Unterwald-Lowndes factorized form:[8,9]

$$\varepsilon_j^L(\omega) = \varepsilon_{\infty,j} \prod_l^k \frac{\omega^2 + \mathrm{i}\gamma_{\mathrm{LO},lj}\omega - \omega_{\mathrm{LO},lj}^2}{\omega^2 + \mathrm{i}\gamma_{\mathrm{TO},lj}\omega - \omega_{\mathrm{TO},lj}^2} \tag{S3.1}$$

with high-frequency limit ε_∞, transverse optical (TO-) and longitudinal optical (LO-) frequencies $\omega_{\mathrm{TO/LO}}$, and respective damping parameters $\gamma_{\mathrm{TO/LO}}$ taken from literature:[10,11]

Table S2: literature data for the dielectric function of LAO (all values in cm^{-1}):

$\omega_{TO,1}$	$\gamma_{TO,1}$	$\omega_{LO,1}$	$\gamma_{LO,1}$	$\omega_{TO,2}$	$\gamma_{TO,2}$	$\omega_{LO,2}$	$\gamma_{LO,2}$	$\omega_{TO,3}$	$\gamma_{TO,3}$	$\omega_{LO,3}$	$\gamma_{LO,3}$
188.0	0.4	276.4	3.7	427.0	5.0	596.1	7.2	495.7	3.8	495.5	3.8
$\omega_{TO,4}$	$\gamma_{TO,4}$	$\omega_{LO,4}$	$\gamma_{LO,4}$	$\omega_{TO,5}$	$\gamma_{TO,5}$	$\omega_{LO,5}$	$\gamma_{LO,5}$	ε_{st}	ε_∞		
650.8	22.5	744.1	12.1	708.2	55.3	702.2	66.0	22.3	4.12		

Table S3: literature data for the dielectric function of STO (all values in cm^{-1}):

$\omega_{TO,1}$	$\gamma_{TO,1}$	$\omega_{LO,1}$	$\gamma_{LO,1}$	$\omega_{TO,2}$	$\gamma_{TO,2}$	$\omega_{LO,2}$	$\gamma_{LO,2}$	$\omega_{TO,3}$	$\gamma_{TO,3}$	$\omega_{LO,3}$	$\gamma_{LO,3}$	ε_{st}	ε_∞
91	15.0	172	3.8	175	5.4	474	4.5	543	17.0	788	25	310	5.1

Figure 1c, 1d, 1f, and following:

Finite dipole model (FDM) parameters were 400 nm ellipsoid length, 90 nm tip radius, 80 nm tapping amplitude, demodulation order $n = 2$, and the geometric factor $g = 0.7\times\exp(0.1\mathrm{i})$. The p-polarized transfer matrix method (TMM) reflection coefficient was used as the FDM sample reflection factor,[12] at a dominant in-plane wavevector[13] of $k_x = 250\,000$ cm^{-1}. The LAO thickness was 8 unit cells (3 nm) and the dielectric function of the 2D electron gas (2DEG) was described by adding a Drude term to the phonon background of STO:[8]

$$\varepsilon_j^{DL}(\omega) = \varepsilon_j^L(\omega) + \varepsilon_j^D(\omega) = \varepsilon_{\infty,j} \prod_l^k \frac{\omega^2 + \mathrm{i}\gamma_{\mathrm{LO},lj}\omega - \omega_{\mathrm{LO},lj}^2}{\omega^2 + \mathrm{i}\gamma_{\mathrm{TO},lj}\omega - \omega_{\mathrm{TO},lj}^2} - \frac{\omega_p^2}{\omega^2 + \gamma^2} + \mathrm{i}\frac{\gamma\omega_p^2}{\omega(\omega^2 + \gamma^2)} \tag{S3.2}$$

Here, ω_p is the plasma frequency and γ is the electron damping parameter, respectively described as:

$$\omega_p^2 = \frac{n_{\mathrm{2D}}}{z_0} \cdot \exp\left(-\frac{z}{z_0}\right) \cdot \frac{e^2}{\varepsilon_0 m^*} \quad \text{and} \quad \gamma = \frac{e}{m^*\mu} \tag{S3.3}$$

for an exponentially decaying carrier concentration.[1] To model the depth distribution of the 2DEG, z is the distance from the LAO/STO interface, μ is the carrier mobility and n_{2D} the sheet carrier density. The latter is transformed into a volume carrier density which then decays exponentially with decay constant z_0 away from the interface. This distance-dependent dielectric function was then used in a multilayer approach of 10 slices with thickness $d = 1$ nm, with an averaged effective mass of $m^* = 3.2\ m_0$. For Figure 1d, the values are $\mu = 2$ cm²/Vs and $n_{2D} = 3\times10^{13}$ cm^{-2}, for Figure 1e and 1f the mobility is enhanced to $\mu = 10$ cm²/Vs.

Figure 2:

Table S4: modelling parameters used in Figure 2, compared to room temperature Hall measurement data of the sample:

	n_{2D} (cm^{-2})	μ (cm^2/Vs)
Hall meas. (T = 300 K)	4.3×10^{13}	5.1
Figure 2a	4×10^{13}	5, 10, 20, 40, 80
Figure 2b	$(2, 3, 4, 5, 6) \times 10^{13}$	20
Figure 2c, *parameter set A*	6×10^{13}	20

Table S5: modelling parameters used in Figure 2c, parameter set B (variation of phonon parameters):

	n_{2D} (cm^{-2})	μ (cm^2/Vs)	ω_{TO} (cm^{-1})	ω_{LO} (cm^{-1})	γ_{TO} (cm^{-1})	γ_{LO} (cm^{-1})
**2DEG**	8×10^{13}	10				
**LAO**			660	780	22.5	8
**STO**			570	785	17	25

Figure 3c:

Table S6: modelling parameters used in Figure 3c:

n_{2D} (cm^{-2})	μ (cm^2/Vs)
4×10^{13}	8, 12, 18, 27, 40

S4: Low-temperature transport

Figure S3 shows electronic transport data obtained in a mimicked Hall bar geometry, for which an approximately 1x5 mm^2-sized stripe was cut from the sample and contacted by wire bonding. Figure S3a shows the temperature-dependent resistance of the samples, while Figure S3b shows the field-dependence of the transversal Hall resistance at various temperature. Below temperatures around 30 K a non-linear Hall resistance is observed, which can be modeled based on a two-band conduction mechanism, typically applied for LAO/STO interfaces[14,15] . The resulting mobilities and carrier densities are displayed in Figures S3c and S3d, revealing a high-mobility-low-density and a low-mobility-high-density electron species, with typical figures for LAO/STO samples. Note that in DC transport the absolute mobility values can differ strongly from mid-infrared mobilities, as explained in the main text.

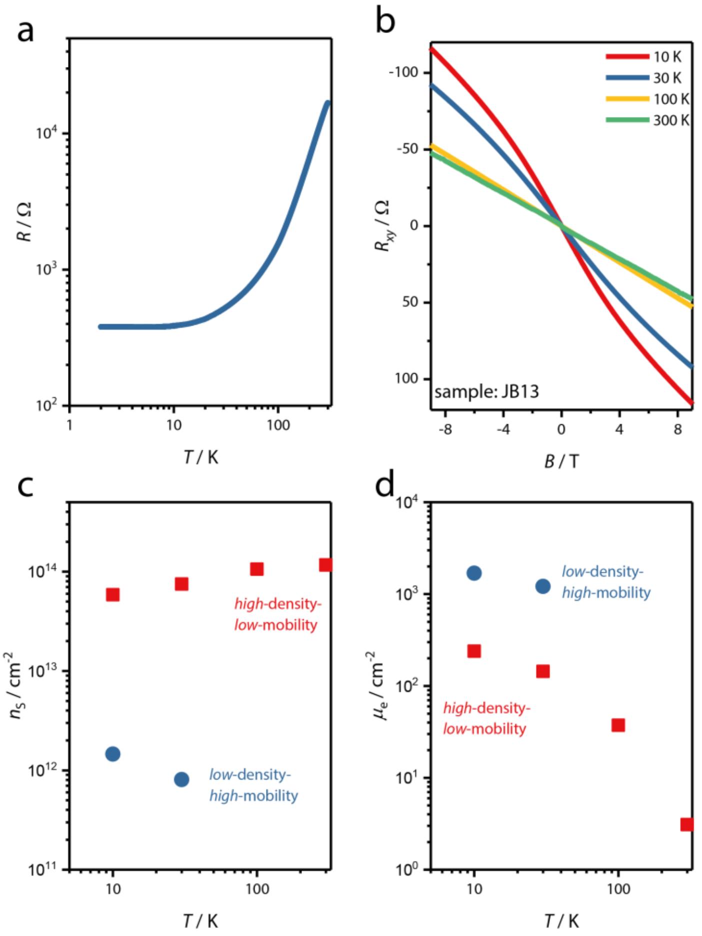

Figure S3: Electronic transport data from mimicked Hall-bar geometry from a 1x5 mm² stripe of the LAO/STO sample. **a** Temperature-dependent resistance and **b** field-dependence of the transversal Hall resistance at various temperatures. **c** The resulting mobilities and **d** carrier densities resulting from assuming a two-band conduction mechanism.

S5: Inhomogeneity and influence of line scan

Figure S4 presents scattering-type scanning near-field optical microscopy (s-SNOM) images of the conducting LAO/STO sample recorded at $\nu = 700\ cm^{-1}$. For these images, an illumination frequency with the *highest overall signal* around the phonon near-field resonance was picked (cf. Figure 1e of the main text), to ensure sufficient signal-to-noise ratio for image recording. At this time, illumination frequencies for *high contrast* between different electronic properties were not yet known, as the fingerprint spectra were calculated afterwards.

As explained in the discussion of Figure 3 of the main text, an inhomogeneity observed in the near-field amplitude (Figure S4a) and the corresponding phase (Figure S4b) was further investigated with a hyperspectral line scan along the dashed line, i. e. sequential spectroscopy by varying the laser frequency between 600 and 950 cm^{-1} at each point along the line. After recording this line scan, another s-SNOM image was recorded in amplitude (Figure S4d) and phase (Figure S4e). While the topography (Figure S4c and S4f) shows that the measurement position was unchanged (indicated by red ovals), the optical image changed drastically in both amplitude and phase. In Figure S4e, a lower phase value (dark blue) is visible along the diagonal from bottom left to top right, following the position of the line scan. This could indicate that the preceding s-SNOM measurement itself is responsible for the observed change in near-field response. Similar effects in low-temperature scanning probe measurements are known from literature and a current topic of research, e.g. arising due to electrostatic gating from the irradiated AFM tip,[1] persistent photoconductivity,[16,17] or frozen condensates.[18] An influence of the preceding measurement is further supported by the fact that the first image (top row) was aborted after ~90% completion (black region at the lower end), which can be seen in the second image (bottom row) as a bright yellow square in the amplitude and a blue square in the phase. Therefore, it can be assumed that the inhomogeneity observed in the first image is itself a result of the preprogrammed movement algorithm of the scanning tip, that is executed when starting and aborting measurements from different positions.

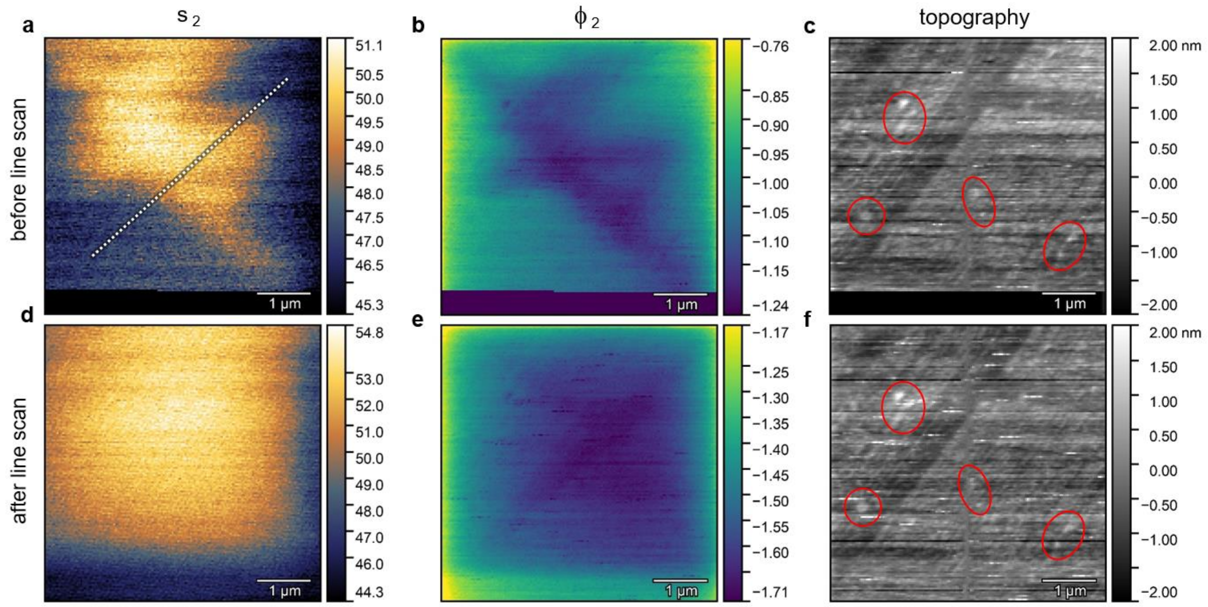

Figure S4: s-SNOM images of near-field amplitude s_2 (a, d), phase φ_2 (b,e) and topography (c,f), measured at $\omega = 700\ cm^{-1}$. Subfigures a-c show the measurement before a hyperspectral line scan was recorded (partially shown in Figure 3b of the Main Text) along the dashed line indicated in Figure S4a. Subfigures d-f show a subsequent measurement, after the hyperspectral line scan was completed. Note that the first measurement (a-c) was aborted after 90% completion, indicated by a dark region at the bottom of each image. Red ovals in the topography highlight characteristic points, showing that position on the sample and scan range are identical.

S6: Measurement parameters

For all measurements:

frequency resolution: 1 cm^{-1}
PsHet modulation frequency: 300 Hz
sample temperature: 8 K

Figure 2c:

Table S7: measurement parameters used in Figure 2c:

parameter	pos 1	pos 2
frequency (cm^{-1})	600 – 950	600 – 950
averaging	2	1
integration time (ms)	98	98
tip frequency (kHz)	237	237
tapping amplitude (nm)	77	80

Figure 3a / S2:

Table S8: measurement parameters used in Figures 3a and S2:

parameter	before linescan	linescan	after linescan
scan size (μm)	5.1 × 5.1	5.6	5.1 × 5.1
scan resolution (px)	150 × 150	20	150 × 150
frequency (cm^{-1})	700	600 – 950	700
averaging	1	2	1
integration time (ms)	20	98	20
tip frequency (kHz)	237	237	237
tapping amplitude (nm)	77	85	85

References

1. Luo, W. *et al.* High sensitivity variable-temperature infrared nanoscopy of conducting oxide interfaces. *Nat. Commun.* **10,** 2774; 10.1038/s41467-019-10672-5 (2019).

2. Barnett, J. *et al.* Phonon-enhanced near-field spectroscopy to extract the local electronic properties of buried 2D electron systems in oxide heterostructures. *Adv. Funct. Mater.* **30,** 2004767; 10.1002/adfm.202004767 (2020).

3. Hermann, P. *et al.* Near-field imaging and nano-Fourier-transform infrared spectroscopy using broadband synchrotron radiation. *Opt. Express* **21,** 2913–2919; 10.1364/OE.21.002913 (2013).

4. Hermann, P. *et al.* Enhancing the sensitivity of nano-FTIR spectroscopy. *Opt. Express* **25,** 16574–16588; 10.1364/OE.25.016574 (2017).

5. Xu, X. G., Rang, M., Craig, I. M. & Raschke, M. B. Pushing the Sample-Size Limit of Infrared Vibrational Nanospectroscopy: From Monolayer toward Single Molecule Sensitivity. *J. Phys. Chem. Lett.* **3,** 1836–1841; 10.1021/jz300463d (2012).

6. Bensmann, S. *Breitband-Nahfeldmikroskopie an phonon-resonanten Kristallen* (PhD thesis, RWTH Aachen University, Aachen, 2016).
7. PT277-XIR series. Single Housing MIR Tunable Picosecond Laser. Available at https://ekspla.com/product/single-housing-mid-ir-range-tunable-picosecond-laser-pt277xir/ (2023).
8. Gervais, F., Servoin, J.-L., Baratoff, A., Bednorz, J. G. & Binnig, G. Temperature dependence of plasmons in Nb-doped $SrTiO_3$. *Phys. Rev. B* **47,** 8187–8194; 10.1103/PhysRevB.47.8187 (1993).
9. Schöche, S. *et al.* Infrared dielectric functions, phonon modes, and free-charge carrier properties of high-Al-content $Al_xGa_{1-x}N$ alloys determined by mid infrared spectroscopic ellipsometry and optical Hall effect. *J. Appl. Phys.* **121,** 205701; 10.1063/1.4983765 (2017).
10. Kamarás, K. *et al.* The low-temperature infrared optical functions of $SrTiO_3$ determined by reflectance spectroscopy and spectroscopic ellipsometry. *J. Appl. Phys.* **78,** 1235–1240; 10.1063/1.360364 (1995).
11. Willett-Gies, T., DeLong, E. & Zollner, S. Vibrational properties of bulk $LaAlO_3$ from Fourier-transform infrared ellipsometry. *Thin Solid Films* **571,** 620–624; 10.1016/j.tsf.2013.11.140 (2014).
12. Hauer, B., Engelhardt, A. P. & Taubner, T. Quasi-analytical model for scattering infrared near-field microscopy on layered systems. *Opt. Express* **20,** 13173–13188; 10.1364/OE.20.013173 (2012).
13. Fei, Z. *et al.* Infrared nanoscopy of dirac plasmons at the graphene-SiO_2 interface. *Nano Lett.* **11,** 4701–4705; 10.1021/nl202362d (2011).
14. Gunkel, F. *et al.* Defect control of conventional and anomalous electron transport at complex oxide interfaces. *Phys. Rev. X* **6**; 10.1103/PhysRevX.6.031035 (2016).
15. Joshua, A., Pecker, S., Ruhman, J., Altman, E. & Ilani, S. A universal critical density underlying the physics of electrons at the $LaAlO_3/SrTiO_3$ interface. *Nat. Commun.* **3,** 1129; 10.1038/ncomms2116 (2012).
16. Eom, K. *et al.* Origin of the giant persistent photoconductivity in $LaAlO_3/SrTiO_3$ heterostructures probed by noise spectroscopy. *J. Mater. Sci. Technol.* **137,** 152–158; 10.1016/j.jmst.2022.08.006 (2023).
17. Irvin, P. *et al.* Rewritable nanoscale oxide photodetector. *Nat. Photonics* **4,** 849–852; 10.1038/nphoton.2010.238 (2010).
18. Bi, F. *et al.* "Water-cycle" mechanism for writing and erasing nanostructures at the $LaAlO_3/SrTiO_3$ interface. *Appl. Phys. Lett.* **97,** 173110; 10.1063/1.3506509 (2010).
